# Glucose status and depressive symptoms: a cohort study of elderly people in northwest Finland

**DOI:** 10.1080/02813432.2019.1608050

**Published:** 2019-05-17

**Authors:** Yrjö Perkkiö, Jari Jokelainen, Juha Auvinen, Pasi Eskola, Juha Saltevo, Sirkka Keinänen-Kiukaanniemi, Markku Timonen

**Affiliations:** a Center for Life Course Health Research, Faculty of Medicine, University of Oulu, Oulu, Finland;; b Health Centre of Muonio and Enontekiö, Muonio, Finland;; c Medical Research Center, Oulu University Hospital, University of Oulu, Oulu, Finland;; d Central Finland Central Hospital, Jyväskylä, Finland

**Keywords:** Diabetes, depression, arctic

## Abstract

**Objective:** To assess the association between depressive symptoms and impaired glucose metabolism in the elderly population in arctic latitudes.

**Design:** A population-based study. *Setting.* Community.

**Subjects:** The study population consisted of 1,830 subjects born between the years 1915 and 1958 in the northernmost part of Finland, the Muonio-Enontekiö district, who participated in a health survey during 1974–1984. In 2014, a health questionnaire was sent to 1,037 subjects, and 757 participants (73%) answered it. Those (*n* = 629) living in the Muonio-Enontekiö district undergone a clinical examination in 2014 and 2015 including blood collections.

**Main outcome measures:** Depressive symptoms defined by the Beck Depression Inventory II (BDI II) with a cut-off point of 14. Different diabetic states based on WHO’s classification criteria defined by fasting plasma glucose and ADA’s criteria by glycosylated haemoglobin (HbA1c) values.

**Results:** According to logistic regression analysis, depressive symptoms (BDI-II ≥ 14) were associated statistically significantly with previously known type 2 diabetes, the odds ratio (OR) being 4.33 (95% CI 1.53–14.14). Regarding prediabetic fasting glucose/HbA1c values, the corresponding OR was 2.94 (95% CI 1.17–8.94). The prevalence of depressive symptoms (BDI-II ≥ 14) was 7.1%, (men 9.7% and women 5.4%) and 13.7% (men 9.9% and women 17.0%) in subjects living in Muonio-Enontekiö district and in those who had moved away from there, respectively.

**Conclusions:** The association of depressive symptoms between prediabetes and diabetes seems to be present also in the northernmost latitudes of the world.

## Introduction

Type 2 diabetes (T2D) is one of the most common and most serious medical conditions in modern healthcare. Furthermore, undiagnosed T2D is as common as diagnosed diabetes, and almost one-fourth of middle-aged people have pre-diabetes [1]. What is more, it has been estimated that the prevalence of T2D will be double after 10–15 years e.g., in Finland (Finnish Current Care Diabetes Guidelines 2016). Consequently, T2D will have increasing economic health care system burden worldwide [2].

T2D is often accompanied by troubling psychiatric conditions such as depression. Indeed, depression is known to be 2–3 times more prevalent in people with T2D compared to those without it [3]; and in clinical practice, it is challenging to treat these diseases simultaneously. In addition, the coexistence of T2D and depression leads to an increased risk of mortality [4–6]. While T2D distress, a consequence of the psychological distress created by the diagnosis, is now widely accepted as one pathophysiological mechanism behind the co-existence between T2D and depression, depression/depressive symptoms are, on the other hand, also known to be associated with previously unknown diabetes and prediabetic states when subjects are not yet suffering from T2D [7–12].

To the best of our knowledge, there are no previous studies about the prevalence of depressive symptoms and its association with the glucose status among people living in the very rural district in the arctic areas of the world. The aim of the present study was to evaluate the association of impaired glucose metabolism and depressive symptoms in the elderly population living in the most northern part of Finland above Arctic Circle.

## Methods

### Study population

The study population consists of persons born between the years 1915 and 1958 in the Muonio-Enontekiö district in the northernmost part of Finland (150–370 kilometres north of the arctic circle) who participated in regular health surveys from the year 1974 to 1984. In 1984, the total number of the cohort consisted of 1,830 subjects.

**Figure 1.**
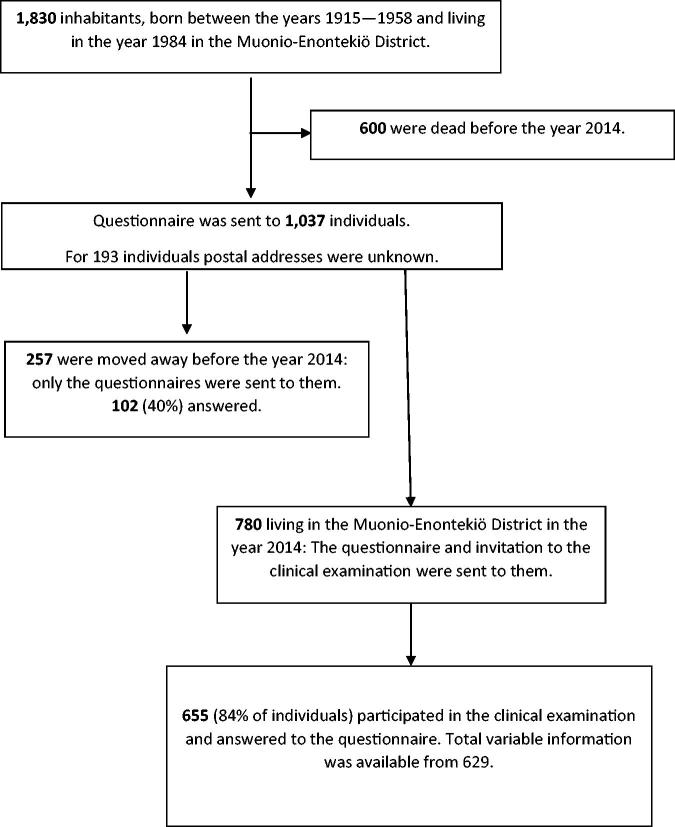
Flow chart of the Muonio-Enontekiö District study population.

In 2014 follow-up survey, the postal questionnaires were sent to 1,037 (600 persons had died, for 193 individuals postal addresses were unknown) of the subjects who participated in the studies from the year 1974 to 1984; Of these, 257 persons (mean age was 72.69 years) had moved away from the Muonio-Enontekiö district; 102 (40%) of those answered the questionnaire. An invitation to clinical examinations was sent along with postal questionnaires to those living in the Muonio-Enontekiö district (*n* = 780), and 655 (84%) of them attended (mean age was 71.62 years). Total variable information was available from 629 (81%) individuals (Figure 1).

Specially trained study nurses conducted the clinical examinations and took the blood samples for laboratory analyses in the Muonio-Enontekiö district during winter 2014–2015.

The Ethics Committee of the Northern Ostrobothnia Hospital District approved the research protocol on 21.1.2013.

### Depressive symptom profiles

The 21-item Beck Depression Inventory-II questionnaire was included into the postal questionnaires. The total score was calculated, and subjects having a total score of ≥14 points were defined having clinically significant depressive symptoms [13].

### Glucose metabolism

Fasting plasma glucose (FPG) and glycosylated hemoglobulin (HbA1c) were performed after an overnight (12-h) fasting period. Glucose status was classified: (1) normal glucose (NGT) was defined as having FPG concentration < 6.1 mmol/l or glycosylated hemoglobulin (HbA1c) < 39 mmol/mol (5.7%); (2) prediabetes was defined as having FPG of 6.1–6.9 mmol/l (IFG) or HbA1c 39–47 mmol/mol (5.7–6.4%); and (3) type 2 diabetes (T2D) was defined as having FPG at least 7.0 mmol/l or HbA1c 48 mmol/mol (6.5%) or more. Previously diagnosed T2D was defined by self-reported diagnosis made by a physician.

### Physical activity

Participants reported their physical activity in a postal questionnaire: Subjects were asked ‘How many times per week was their physical activity at least 30 minutes per day’. In the statistical analyses, answers were dichotomized so that ‘physical activity’ was defined to be present if subject reported having had ‘Five times or more’ ‘physical activity at least 30 minutes per day’.

### Weight and height

In the clinical examinations, participants (without clothes and shoes) were weighed (kg) using a digital scale. Height (cm) was measured (participants without shoes, wall scale) by a nurse. Body mass index (BMI) was calculated (kg/m^2^) based on measured height and weight and classified by the WHO definition (underweight BMI <18.5, normal weight BMI 18.5–24.9, overweight BMI 25–29.9, obese BMI 30–34.9, severe obesity 35–39.9 and very severe obesity BMI >40 kg/m^2^).

### Antidepressant use

Antidepressant use was based on a self-reported list of medication.

### Statistical analyses

Data are presented as means and standard deviations or in proportions, unless otherwise indicated. The Kruskal-Wallis test was used to analyse the difference in characteristic variable distributions for continuous variables, while Pearson’s chi-square test was used for categorical variables. We estimated the prevalence of depressive symptoms using inverse probability weighting (IPW) methods that adjusted for non-responses. We calculated response probability by sex, age and place of residence, using a logistic regression model. The weight of each subject was given by the inverse of the predicted probability. We used logistic regression analysis to examine the association of glucose metabolism and depressive symptoms. Unadjusted and adjusted odds ratios (OR) and their 95% confidence interval (95% CI) are presented. Statistical analyses were performed using SAS 9.4 for WINDOWS, and *p* < .05 was considered statistically significant.

## Results

### Characteristics of the study population

Mean age of men was 71.9 (7.7) years and of women 70.5 (8.8) years; the detailed description of the whole study population is presented in Table 1.

**Table 1.** Characteristics of the study subjects (*n* = 629).

	Men	Women
	*N* = 249	*N* = 380
Age, mean (sd)	72.3 (7.7)	71.2 (8.8)
BMI, mean (sd)	26.9 (3.8)	27.7 (4.7)
FPG (mmol/L), mean (sd)	6.5 (1.3)	6.5 (1.6)
Normal (<6.1 mmo/l), N (%)	78 (31.3)	174 (45.8)
Prediabetes (6.1-6.9 mmol/l), N (%)	111 (44.6)	123 (32.4)
Type 2 diabetes (≥7.0 mmol/l), N (%)	60 (24.1)	83 (21.8)
HbA1c (mmol/l), mean (sd)	40.2 (7.5)	41.0 (9.0)
HbA1c (%), mean (sd)	5.8 (0.7)	6.1 (3.4)
Normal (<5.7%), N (%)	122 (49.0)	180 (47.9)
Prediabetes (5.7–6.4%), N (%)	103 (41.4)	146 (38.8)
Type 2 diabetes (≥6.5%), N (%)	24 ( 9.6)	50 (13.3)
Glucose status, N (%)		
Normal	58 (23.3)	128 (33.7)
Prediabetes	127 (51.0)	159 (41.8)
Type 2 diabetes	32 (12.9)	28 ( 7.4)
Previous diagnosed T2D	32 (12.9)	65 (17.1)
Low education, N (%)	205 (85.4)	279 (75.0)
Smoking, N (%)	27 (11.1)	38 (10.1)
Hypertension, N (%)	124 (51.7)	194 (52.3)
Physical activity (%)		
No physical active due to of disability	16 ( 6.7)	20 ( 5.4)
1–4 times per week	87 (36.2)	96 (25.8)
≥5 times per week	137 (57.1)	256 (68.8)
Alcohol consumption (audit), mean (sd)	4.0 (4.6)	1.3 (2.5)
≥ 8	39 (16.5)	12 (3.3)
BDI-II, N (%)		
Mild, ≥14	23 (9.2)	20 (5.3)
Moderate, ≥20	14 (5.6)	18 (4.7)

BMI: body mass index; FPG: Fasting plasma glucose; HbA1c: glycosylated haemoglobin; Audit: alcohol consumption questionnaire; BDI: Beck Depression Inventory-II questionnaire; sd: standard deviation.

**Table 2.** The prevalence of depressive symptoms (BDI-II ≥14 points) among those men and women living still in Muonio-Enontekiö district at 2014 and those who had moved away from area.

	Depressive symptoms (BDI-II ≥14 points)	
	*N*	Weighted % (95% CI)^a^
Men		
All men	28	9.7 (6.1–13.3)
Those living still in Muonio-Enontekiö district	24	9.7 (6.0–13.4)
Those who had moved away	4	9.9 (0.3–19.5)
**Women**		
All women	31	8.1 (5.2–11.0)
Those living still in Muonio-Enontekiö district	21	5.4 (3.2–7.7)
Those who had moved away	10	17.0 (7.1–26.9)

^a^Percentages and 95% confidence intervals are weighted by participation, gender, age and area

BDI-II^b^ = Beck Depression Inventory-II questionnaire.

CI = confidence interval.

Most of the participants were overweight (42.2%), 21.7% were obese, only 30,4% were of normal weight and 1.1% were underweight. Altogether, 3.7% were severe obese and 1.0% were very severe obese. Seven percent of the participants reported having had no physical activity at all because of injury or sickness, and 61% of participants exercised ‘daily at least 30 minutes and 5 times per week’. Finally, antidepressant medication was used by 5.3% of the participants.

The prevalence of mild depression (BDI II at least 14 points) with subjects living still in the Muonio-Enontekiö district was 7.1%. Interestingly, depressive symptoms tended to be more common in men (9.7%) than in women (5.4%). The prevalence of moderate depression (BDI >20) among men was 4.6% and women 4.7%.

Of those who had moved away from the Muonio-Enontekiö district, 102 subjects returned the BDI II questionnaire. The prevalence of depressive symptoms among them was 13.7% (men 9.9% and women 17%) (Table 2); The prevalence of self-reported physician-diagnosed T2D was 15.4% (men 12.9% and women 17.1%).

Among individuals living in the Muonio-Enontekiö district, 40.0% (*n* = 252) had a normal FPG level (men 31.3%, women 45.8%), and 48.0% (*n* = 302) (men 49.0%, women 47.9%) had a normal HbA1c level. The prevalence of IFG was 37.2% (*n* = 234) (men 44.6% and women 32.4%) according to FPG, and 39.7% (*n* = 249) (men 41.4%, women 38.8%) according to HbA1c. T2D was present in 22.8% (*n* = 143) (men 24.1%, women 21.8%) and in 11.8% (*n* = 74) (men 9.6%, women 13.3%) of the subjects according to FPG and HbA1c, respectively. The prevalence of self-reported previously diagnosed T2D was 15.2% (*n* = 97) (12.9% men and 17.1% women).

### Depressive symptoms and glucose metabolism

The prevalence of depressive symptoms (BDI-II ≥ 14) was 13.0% (95% Cl 6.8–19.2) and 6.3% (95% Cl 4.2–8.3) in the participants with previously diagnosed and undiagnosed T2D, respectively; the difference was statistically significant (p 0.0119). The corresponding prevalence was 9.1% and 9.5% in the participants with diabetic FPG and HbA1c values, respectively, while the prevalence of depressive symptoms was 8.1% and 7.6% in the participants with prediabetic FPG and HbA1c values, respectively. Only 2.7% of the participants with normal FPG and HbA1c values had depressive symptoms.

**Figure 2.**
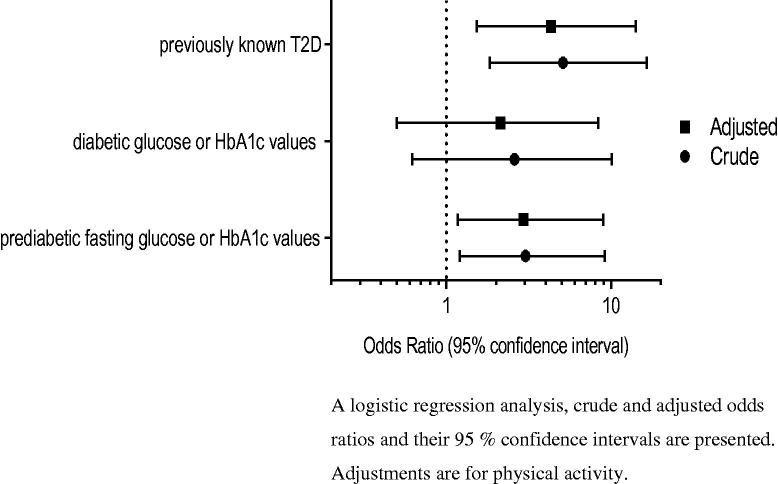
Forest plots showing the OR and 95% CI of depression with different glucose metabolism status.

According to logistic regression analysis, depressive symptoms (BDI-II ≥ 14) were associated statistically significantly with previously known T2D, the odds ratio OR being 4.33 (95% CI 1.53–14.14). Regarding prediabetic fasting glucose or HbA1c values, the corresponding OR was 2.94 (95% CI 1.17–8.94) (Table 3). Depressive symptoms did not associate significantly with diabetic glucose or HbA1c values of those without previously diagnosed T2D: OR 2.13(95% CI 0.50–8.43). The association of depressive symptoms between both previously known T2D and prediabetic fasting glucose of HbA1c values remained statistically significant after adjusting for physical activity (Figure 2).

**Table 3.** Dependent variable: Depressive participants (BDI at least 14): excluded those who moved away from the Muonio-Enontekiö district.

crude	
All	
Prediabetic fasting glucose	2.94 (1.17, 8.94)
or HbA1c values	*p*=.04
Diabetic fasting glucose	2.13 (0.50, 8.43)
or HbA1c values	*p* = 0.28
Previously known T2D	4.33 (1.53, 14.14)
	*p*=.01
Physical activity 30 min	0.48 (0.20, 1.28)
daily 0–4 times/week	*p*=.13
Physical activity 30 min	0.22 (0.09, 0.57)
daily at least 5 times	*p*=.002
Normal	Reference
Observations	629
Log Likelihood	−145.90
Akaike Inf. Crit.	303.80

^a^Beck Depression Inventory-II questionnaire.

^b^Glycated haemoglobin A1c.

^c^Type 2 Diabetes.

## Discussion

### Statement of principal findings

In the present study, we investigated the coexistence of depressive symptoms with glucose metabolism among elderly subjects living in the northernmost part of Finland. The prevalence of previously known T2D was two times higher in those with depressive symptoms than in those without depressive symptoms. Furthermore, prediabetic glucose and HbA1c values were associated with higher prevalence of depressive symptoms. Those with diabetic glucose and HbA1c values had more depressive symptoms than those with normal values. The prevalence of depressive symptoms was three times higher in women who had moved away from the Muonio-Enontekiö district. Participating actively in physical activity was two times more prevalent among those without depressive symptoms than among those with depressive symptoms.

### Strengths and weaknesses of the study

The strength of the present study is the high participation rate, which was 84% of those living in the Muonio-Enontekiö district. Unfortunately, the participation rate was low among those subjects who had moved away (40%). The strengths of our study are that our sample consists of about 60% of the population aged 65 or older and living in the same district in 2014. In 2014, a total of 4,257 inhabitants were living in the Muonio-Enontekiö district (0.4 inhabitants/km^2^). Thus, the people of this cohort are living in the rural district where the climate is cleanest in Europe (WHO’s follow up-report in the years 2008–2014) and winter lasts over half of a year. The main limitation of the present study is that we could not make the clinical examinations for those who had moved away from the Muonio-Enontekiö district.

Unfortunately, we did not have possibility to perform oral glucose tolerance test (OGTT). The reason was long distances and not enough resources. According to previous studies OGTT would have diagnosed more T2D than using only fasting glucose and HbA1c test [14].

### Findings in relation to previous studies and meaning of the study

Our results regarding previously diagnosed T2D is in line with earlier studies showing 1.6 to 2 times higher prevalence of depression symptoms among diabetics [15]. Our findings regarding the association of depressive symptoms between prediabetic glucose and HbA1c values also parallel the earlier meta-analysis concerning the cross-sectional association observed between depression and insulin resistance [7]. We consider our results from the northernmost latitudes to be important, since it was earlier shown that environmental factors that are unique to individuals might contribute to the co-occurrence of depression and type 2 diabetes in midlife [16]. Thus, investigations in different geographical areas and among different ethnic groups are important. People moving within the same culture, for instance from rural to urban areas, also feel the stress of migration, change of environment and altered social support. All these factors tend to interact and to increase stress which, according to the stress diathesis model, is likely to increase the likelihood of mental illness [17]. Our study found that the proportion of females with depressive symptoms was three times higher in those who had moved away from the Enontekiö-Muonio district compared to those who had stayed in the area. Furthermore, the predominance of depressive symptoms in men who had stayed in the Muonio-Enontekiö district was another surprising finding in our study. In general, women are shown to experience depression at twice the rate of men [18]. We do not have the explanation for this unexpected gender difference, which remains to be further studied as also the question of why women who had moved away from the Muonio-Enontekiö district had a very high prevalence of depressive symptoms.

In Finnish Health Study 2011 the prevalence of self-reported diabetes with men (aged 65–74) were 19.5% and women 14.4% [19]. Our results were nearly in the same level.

We did not find any study of the prevalence of depressive symptoms with elderly people above Arctic Circle. We found only studies of the seasonal affective disorders (SAD, atypical depression). The prevalence of SAD was 9% with adult people in Fairbanks in Alaska [20].

Many studies have concluded that depression observed among people with diabetes would be the result of psychological distress created by the disease (diabetes distress), but it is not the only reason for depressive symptoms in people with T2D [1,9–12,21,22]. However, the prevalence of diabetes distress is relatively high, and common in people with depressive symptoms [23]. Thus, in clinical practice, it is important to pay attention to the psychological symptoms of patients with T2D to explore the possible co-existence of depression, and on the other hand, to be careful not to over-diagnose depression. Regarding the bidirectional association between type 2 diabetes and depression, it has already been shown earlier that depression is associated with a 60% increased risk of type 2 diabetes, while type 2 diabetes is only modestly associated with increased risk of depression [24].

In our study, there were only 34 participants who had antidepressant medication, and only two of them had significant depressive symptoms. Unfortunately, we could not analyse the associations between antidepressant use and T2D or glucose metabolism due to this low number of subjects. A Danish study showed that people with diabetes are 65% more likely to fill prescriptions for antidepressants than those without diabetes [25]. The increased risk of an antidepressant prescription is more marked in men with diabetes than in corresponding women. In addition, the increased risk of an antidepressant prescription seen in people with diabetes is more marked in people with lower incomes. Consequently, it could be theorized that intervention to prevent depression after a diagnosis of diabetes may need to be targeted more towards men and persons with low-income jobs.

Our data was collected in the area where many people are still living by herding reindeers, hunting, fishing, gathering berries and making firewood. Consequently, in the past, they had plenty of physical activity just getting enough food. We suppose that today, people of this cohort do not have as much physical activity as before, and, therefore, they are more obese than before. It can also be hypothesized that they have more genetic insulin resistance like many other native populations [26].

## Conclusions

We were able to bring new information to the earlier literature concerning the issue under investigation. The association of depressive symptoms between prediabetes and diabetes seems to be present also in the northernmost latitudes of the world. Moving away from a rural area may increase the risk of depressive symptoms among women, but this should be evaluated in more detail in further studies.
